# Role of adaptin protein complexes in intracellular trafficking and their impact on diseases

**DOI:** 10.1080/21655979.2021.1982846

**Published:** 2021-10-21

**Authors:** Juhyun Shin, Arti Nile, Jae-Wook Oh

**Affiliations:** Department of Stem Cell and Regenerative Biotechnology and Animal Resources Research Center, Konkuk University, Seoul, Republic of Korea

**Keywords:** Adaptin protein, clathrin, cargo protein, intracellular trafficking, cellular pathology, virus infection, cancer

## Abstract

Adaptin proteins (APs) play a crucial role in intracellular cell trafficking. The ‘classical’ role of APs is carried out by AP1‒3, which bind to clathrin, cargo, and accessory proteins. Accordingly, AP1–3 are crucial for both vesicle formation and sorting. All APs consist of four subunits that are indispensable for their functions. In fact, based on studies using cells, model organism knockdown/knock-out, and human variants, each subunit plays crucial roles and contributes to the specificity of each AP. These studies also revealed that the sorting and intracellular trafficking function of AP can exert varying effects on pathology by controlling features such as cell development, signal transduction related to the apoptosis and proliferation pathways in cancer cells, organelle integrity, receptor presentation, and viral infection. Although the roles and functions of AP1‒3 are relatively well studied, the functions of the less abundant and more recently identified APs, AP4 and AP5, are still to be investigated. Further studies on these APs may enable a better understanding and targeting of specific diseases.

APs known or suggested locations and functions.

## GRAPHICAL ABSTRACT

**Figure:**
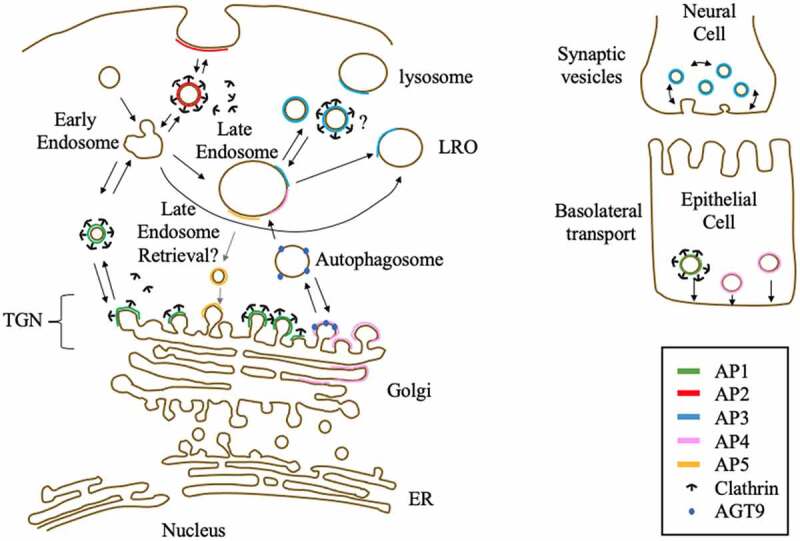

## 1. Introduction

**Figure 1.**
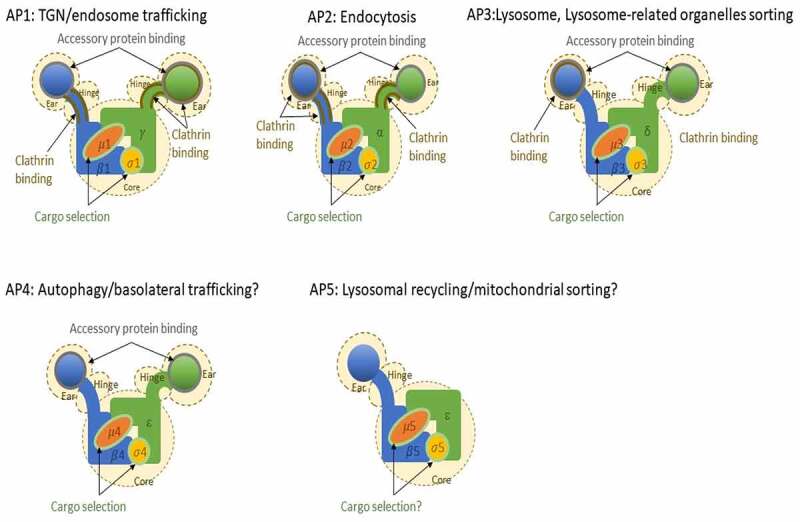
AP complexes with their known or suggested role in cells. Known interaction domains are highlighted

Clathrin adaptor proteins or adaptin proteins (APs) are membrane-bound heterotetrameric complexes localized in cellular buds and vesicles. In addition to clathrin, a structural protein that forms a lattice of hexagons and pentagons around vesicles [1], APs play an important role in intracellular trafficking in the trans-Golgi network (TGN) and beyond. Accordingly, APs are highly conserved and ubiquitously expressed in eukaryotic organisms. Although membrane trafficking from the endoplasmic reticulum to the Golgi is relatively simple and only requires two heptameric coatomer complexes [coat protein type I and II (COPI and COPII)] that form a spherical cage around the membrane to form a vesicle [2], post-TGN trafficking is more complex, as it requires sorting and trafficking to diverse organelles, plasma membranes, and cell specificity. Therefore, post-TGN trafficking requires five APs, several membrane-bound proteins, accessory proteins, and clathrin [3]. The most abundant APs (AP1, AP1, and AP3) and two subunits of the COPI complex share a common ancestral origin [4] and have been identified in various organisms, including model organisms such as *D. melanogaster, C. elegans, S. cerevisiae, S. pombe, H. sapiens, M. musculus*, and *A. thaliana* [5]. Shortly after their discovery, a fourth AP, AP4, was identified [6,7] and detected in various organisms, including the model organisms *H. sapiens, M. musculus*, and *A. thaliana* [5]. However, unlike AP1‒3, AP4 was not found in *D. melanogaster, C. elegans*, and *S. cerevisiae* [8]. AP5, the fifth and last identified complex, has been detected in *H. sapiens* [9] and putatively in *A. thaliana*; however, this AP seems to be lost in several organisms [9]. Identification of an ancestral TSET complex in *Dictyostelium* suggests that eukaryotic AP1‒5s and COPI evolved from a common ancestral complex [10]. All five complexes consist of two large subunits (one common ***β*** unit and the other with a specific letter *γ, α, δ, ε*, or *ζ*), one middle-size subunit (μ) and one small subunit (σ). AP subunits share an estimated sequence similarity of 20 to 80% and have common domains, which were used for their identification [9]. Based on the structures of AP1 and AP2 complex, these complexes were found to consist of (1) a core structure, that include the N-terminal α-helix domains of the two large subunits and the two smaller subunits, which role is to recognizes cargo and localizes the complex in the membrane; and (2) a hinge plus ear structure consisting of the C-terminal domains of the two large subunits, which recognizes clathrin and accessory proteins [11], except for AP5. AP5 lacks a hinge and ear structure in its large ζ subunit, a deletion that results in the loss of cargo and clathrin binding motif (Figure 1). AP subunits have isoforms that are encoded by different genes or by alternative splicing, adding more complexity to the AP complexes (Table 1). Each of the five APs, as well as some of their isoforms, has specific roles and subcellular localization. AP1 and AP2 are the more ‘classic’ APs that interact with clathrin in the TGN (AP1) or plasma membrane (AP2) to form clathrin-coated vesicles and control intracellular trafficking in the TGN and endocytosis, respectively. The location and function of AP3 are less well defined compared to those of AP1 and AP2. AP3 has been detected in the cytosol, TGN, late endosome, lysosomes, and lysosome-related-organelles (LROs) and can interact with clathrin [12]. However, previous research shows that the function of AP3 can be clathrin independent [13]. AP3 is involved in protein sorting of yeast vacuole and human cell lysosome [14] and iin protein trafficking to the late endosome/lysosome [15]. Further, it has two major isoforms that differ in expression site; AP3A is ubiquitously expressed, while AP3B is brain specific [16,17]. Both AP4 and AP5 are relatively less abundant compared to AP1‒3 (subunit concentration is less than 40-fold in HeLa cells [18]) and are not reported to interact with clathrin. As mentioned above, AP subunits share an estimated similarity of 20–80%. For AP1‒3, the estimated sequence similarity is 60 to 90%, while AP4 subunits similarity to the subunits of other complexes ranges from 17 to 43%. Of note, AP4 lacks clathrin-binding domains and is associated with non clathrin-coated vesicles in TGN [7]. AP4 localizes in the Golgi, TGN, and endosomes [6,7,19] and presumably controls autophagy via intracellular trafficking of AGT9, a key autophagosome protein [20], and its accessory protein, Tepsin [21]. These proteins are localized with cation-independent mannose 6-phosphate receptor (CI-MPR), a transmembrane glycoprotein targeting cargo proteins to the lysosome [19], suggesting that AP4 has a role in lysosome sorting. Furthermore, AP4 was shown to be involved in clathrin-independent basolateral signals of epithelial cells [22]. Although AP5 has the same subunits that share sequence and domain similarities with other APs, it lacks a hinge domain and an ear domain and does not have a clathrin-binding site. While AP5 role is yet not well defined, AP5 was found to be localized in the TGN and late endosome and is speculated to function in late endosome retrieval [9]. This speculation is reinforced by findings in a mouse model, where the deletion of AP5 cause a defect in late endosomal retrieval and *in vitro* experiments that demonstrated that mutant cells had defective autophagic functions [23].

**Table 1.** The identified AP complexes

Complex	Subunit	Protein name	Gene name	Gene aliases	Location in human genome (hg38)
**AP1**	Large chain1	γ1	*AP1G1*	*ADTG, CLAPG1*	chr16:71,729,000–71,808,834
		γ2	*AP1G2*	*G2AD*	chr14:23,559,565–23,568,070
	Large chain2	β1	*AP1B1*	*ADTB1, AP105A, BAM22, CLAPB2, KIDAR*	chr22:29,328,821–29,423,179
	Medium chain	μ1A	*AP1M1*	*AP47, CLAPM2, CLTNM, MU-1A, mu1A*	chr19:16,197,911–16,245,906
		μ1B	*AP1M2*	*AP1-mu2, HSMU1B, MU-1B, MU1B, mu2*	chr19:10,572,671–10,587,312
	Small chain	σ1A	*AP1S1*	*AP19, CLAPS1, EKV3, MEDNIK, SIGMA1A*	chr7:101,154,476–101,161,276
		σ1B	*AP1S2*	*DC22, MRX59, MRXS21, MRXS5, MRXSF, PGS, SIGMA1B*	chrX:15,825,806–15,854,813
		σ1 C	*AP1S3*	*PSORS15, sigma1C*	chr2:223,755,326–223,837,582
**AP2**	Large chain1	αA	*AP2A1*	*ADTAA, AP2-ALPHA, CLAPA1*	chr19:49,767,001–49,807,114
		αC	*AP2A2*		chr11:925,870–1,012,240
	Large chain2	β2	*AP2B1*	*ADTB2, AP105B, AP2-BETA, CLAPB1*	chr17:35,587,322–35,726,413
	Medium chain	μ2	*AP2M1*	*AP50, CLAPM1, MRD60, mu2*	chr3:184,174,689–184,184,091
	Small chain	σ2	*AP2S1*	*AP17, CLAPS2, FBH3, FBHOk, HHC3*	chr19:46,838,167–46,850,846
**AP3**	Large chain1	δ	*AP3D1*	*ADTD, HPS10, hBLVR*	chr19:2,100,988–2,151,566
	Large chain2	β3A	*AP3B1*	*ADTB3, ADTB3A, HPS, HPS2, PE*	chr5:78,002,326–78,294,698
		β3B	*AP3B2*	*DEE48, EIEE48, NAPTB*	chr15:82,659,281–82,709,875
	Medium chain	μ3A	*AP3M1*		chr10:74,120,255–74,150,828
		μ3B	*AP3M2*	*AP47B, CLA20, P47B*	chr8:42,152,946–42,171,183
	Small chain	σ3A	*AP3S1*	*CLAPS3, Sigma3A*	chr5:115,841,935–115,914,081
		σ3B	*AP3S2*	*AP3S3, sigma3b*	chr15:89,830,599–89,893,994
**AP4**	Large chain1	ε	*AP4E1*	*SPG51, STUT1*	chr15:50,908,683–51,005,895
	Large chain2	β4	*ap4b1*	*BETA-4, CPSQ5, SPG47*	chr1:113,894,194–113,904,799
	Medium chain	μ4	*AP4M1*	*CPSQ3, MU-4, MU-ARP2, SPG50*	chr7:100,101,664–100,110,319
	Small chain	σ4	*AP4S1*	*AP47B, CLA20, CLAPS4, CPSQ6, SPG52*	chr14:31,025,106–31,096,449
**AP5**	Large chain1	ζ	*AP5Z1*	*KIAA0415, SPG48, zeta*	chr7:4,775,623–4,794,397
	Large chain2	β5	*AP5B1*		chr11:65,773,898–65,780,976
	Medium chain	μ5	*AP5M1*	*C14orf108, MUDENG, Mu5, MuD*	chr14:57,268,971–57,298,742
	Small chain	σ5	*AP5S1*	*C20orf29*	chr20:3,820,547–3,828,838

In this paper, we sought to review the findings reported for each AP subunit, including relatively recent discoveries, while focusing on the pathological involvement of AP complexes.

## 2. AP1 complex

AP1 is an indispensable protein complex in cells as it is known that its main role is in clathrin dependent protein transportation from the TGN to endosome, while evidences show it is also involved in basolateral transport from the TGN in epithelial cells (Figure 1) [24]. Accordingly, its knock-out is embryonically lethal and its missense or nonsense mutations in mouse model organisms result in a serious developmental deficiency [25,26]. In humans, there are two isoforms of the large subunit γ, AP1G1 and AP1G2, and one isoform of the other large subunit β, AP1B1. AP1G1 and AP1G2 are 60% identical. However, AP1G1 is ubiquitously expressed, while AP1G2 is only expressed in plants and vertebrate, with presumably diverse functions and/or stages of expression during development [27]. Based on inhibitor analysis, AP1G1 and AP1G2 might recruit distinct accessory proteins [28] while being indispensable for development [29]. Human AP1G1 variants lead to neurodevelopmental deficiency [26], high risks of pancreatic cancer [30] and cardiac arrest among patients with coronary artery disease [31]. Although required as intracellular trafficking components, AP1G1 and AP1B1 are cofactors that can be hijacked by coronaviruses (SARS-CoV-2, MERS-CoV, and seasonal HCoVs) [32]. Upon HIV infection, AP1G2, but not AP1G1, is used to remove host CD4 receptors [33], which indicates that viruses selectively target the host AP1 complex. In renal cancer, AP1G1 expression is downregulated in patient tumor tissues and suppresses cancer cell proliferation and migration [34]. Upon treatment with cetuximab, an approved anti-cancer drug for head and neck squamous cell carcinoma (HNSCC), membrane-bound epidermal growth factor receptor (EGFR) and alanine-serine-cysteine transporter 2 (ASCT2) were found to interact with AP1G1, resulting in the sensitization of cancer cells to reactive oxygen species (ROS) treatment, which might contribute to a higher survival rate in the patient group showing a higher AP1G1 expression [35]. Taken together this suggest that AP1G1, or AP1 complex might play a role in an intrinsic defense mechanim against cancer. There are two isoforms of AP1 middle-sized subunit μ, AP1M1 that is ubiquitously expressed and AP1M2 that is expressed in polarized epithelial and exocrine cells [36], which specifically function in basolateral transport [37]. Interestingly, as the knock-out of any subunit of AP1 results in AP1 inactivation [38,39] knock-out of one subunit are usually used to study the AP1 complex function [40–42], but two independent studies using forward genetic screening for mutations in cell trafficking detected AP1M1 while, other AP1 subunits were not [41,43]. The ubiquitous AP1M1 subunit is required as it contain the binding domains that recognize proteins andrecruit clathrin [41,44]. However, upregulation of AP1M1 was reported to have pathological consequences; hepatitis B virus, the major cause of liver cancer, was found to cause upregulation of AP1M1 expression, which upregulated cancer cell proliferation [45]. In addition, upregulation of AP1M1 is putatively a biomarker for metastasis to the brain tissue [46]. On the other hand, AP1M2 containing AP1 complex was recently proposed to control β1 integrin transport in the basal membrane of epithelial cells and act as a cell-inherent anticancer mechanism that can inhibit metastasis [47]. There are three AP1 σ subunits: AP1S1, AP1S2, and AP1S3. To date, no reports have been published regarding the cell-specific expression of AP1S1, AP1S2, and AP1S3. Further, each of the three proteins is expressed ubiquitously in human tissues at low levels, with slight enrichment in the brain, epididymis, and a group of tissues (ductus deferens, epididymis, and seminal vesicle) (http://www.proteinatlas.org). However, these subunits also contribute to the complexity of the AP1 complex. For example, during development, AP1S1 and AP1S2 form AP1 complexes with divergent functions in neuronal endosome maturation, that is AP1S1 containing AP1, which binds to the ArfGAP1-Rabex-5 complex, is inhibited by AP1S2 containing AP1 complex, thereby controlling its level [48]. AP1S1 knockdown is embryonically lethal, and in cells, AP1S1 knock-down result in failure of recycling in TGN from endosomes [38] and failure of the targeting low-density lipoprotein-derived cholesterol and glycosphingolipids to late endosome/lysosome [49]. On the other hand, a recent study using bioinformatics have shown that AP1S1 expression is related to glioblastoma multiforme (GBM) pathogenesis [50] and is hypo-methylated and upregulated in acute myeloid leukemia (AML) [51] indicating that its upregulation might have pathological effects in cancer. Similarly, to AP1S1 deficiency, AP1S2 deficiency is linked to failure in the recycling of endosome vesicles and has severe developmental effects [52]. Further, similarly to AP1S1, the main function of AP1S2, which is highly expressed in neurons, is to recognize similar cytosolic domains of proteins for intracellular transport to endosome/lysosome, and exhibit some specificity, for example, sortilin [53]. As AP1S2 is linked to the X-chromosome, its defect is reported to be the cause of X-chromosome-linked mental disease in patients [54]. In addition, AP1S2 downregulation inhibits cancer cell mobility in melanoma cells, suggesting that its regulation might be a target for treatment [55,56]. Mutations in AP1S3 cause pustular psoriasis, an immune disease in skin tissue, via the translocation of Toll-like receptor-3 [57] and disruption of keratinocyte autophagy, resulting in the upregulation of interleukin (IL)-1 signaling [58]. Finally, similarly to that of AP1S2, the upregulation of AP1S3 was found to be linked with cancer cell aggressiveness among breast cancer patients and could be a target for small RNA silencing [59], which highlights its potential as a target for cancer treatment.

## 3. AP2 complex

AP2 is primarily involved in endocytosis from the plasma membrane, and similarly to AP1, its depletion is embryonic lethal in mouse model, showing it is necessary for proper development [60]. However, AP2 is not necessary for clathrin-dependent endocytosis, as it is partially redundant with other clathrin-associated complexes in the endocytosis pathway (Figure 1)[61,62]. AP2 complex has relatively fewer isoforms compared to AP1 and AP3, with only two isoforms of its large α subunit (AP2A1 and AP2A2), while the other subunits are unique [the other large β subunit (AP2B1), medium μ subunit (AP2M1), and small σ subunit (AP2S2)]. AP2A1 recognizes and binds to cargo proteins, clathrin, and accessory proteins, and thus plays a key role in the clathrin-dependent endocytosis process [63–65]. AP2A1 was reported to be involved in neural cell development via binding to m-Numb [66], in hematopoietic stem cell differentiation [67], in the synaptic vesicle cycle pathway and related neurodegenerative diseases [68], in cell defense (e.g., in the antifungal defense mechanism by binding to MYO1F) [69] and in inhibition of HIV genomic incorporation [70]. However, this clathrin endocytosis-related subunit can also serve as a target of viral infection, for example, by being hijacked by HAdV-D37 [71] and HEV71 [72] viruses. Interestingly, in *A. thaliana*, endocytosis was found to involve AP2A1, whereas the uniquely known AP2 middle-sized subunit, AP2M1, was not required, suggesting that other isoforms of AP2 are yet to be discovered [73]. AP2A1 is a candidate biomarker gene among patients with ovarian cancer [74], pediatric medulloblastoma [75], and Alzheimer’s disease [76,77], suggesting that its aberrant expression can be pathological. Both AP2A1 and AP2A2 were reported to confer resistance to erlotinib, an anti-cancer drug, in lung cancer cells [78]; interact with Shc, an SH2-containing proto-oncogene involved in growth factor signaling in mammalian cancer cells [79]; and function in hemopoietic stem cell development [80]. AP2A2 has a partially non-redundant function with AP2A1 owing to its unique peroxisome proliferator-activated receptor (PPAR) α responding domain, which increases fatty acid oxidization in the adipose tissue [81] by interacting with syndecan-2, a mediator of extracellular matrix (ECM) signaling in cardiac tissue [82]. Listeria monocytogenes bacteria-induced cytotoxicity was reportedly inhibited by the binding of bacterial proteins to AP2A2 in a pathway that enable bacterial growth in host cells [83]. Interestingly, unlike AP2A1, no reported correlation was found between AP2A2 and cancer. However, with or without AP2A1, AP2A2 aberrant expression is linked to Alzheimer’s disease [76,77,84–87] and is specifically linked to obesity [81,88], coronary disease [89], chronic bronchitis [90,91] and nicotine withdrawal [92], suggesting that α isoforms may have distinct roles in cells. AP2B1, the other large β subunit of the AP2 subunit, shares common binding proteins with the α subunits. For example, both AP2B1 and AP2A1 bind to full-length IL-33 [93] and both were reported to be involved in common functions, such as synaptic vesicle cycle [9,68,94] and cell development [95,96]. Moreover, the pathological consequences of AP2B1 mutation are similar to those of the AP2α subunit mutation and has been shown to have various effects in neural tissue. In fact, AP2B1 affects dendrite cell morphology by controlling the mTOR pathway [97] through its action as a biosignature upon antidepressant treatment [98], binding to Dynamin-1, and triggering the process of autophagy, which inhibits dementia [99] and is putatively linked to depressive disorder [100]. AP2B1 is also linked to Alzheimer’s disease [101], which explains the importance of the AP2 complex in controlling autophagy and lysosomal protein degradation that regulate this neuronal degenerative disease. AP2B1 was shown to interact with ANO7 in prostate cancer patients, putatively promoting cancer progression [102], increasing chemotherapy resistance in ovarian cancer patients [103], upregulating cancer cell metastasis ability [104], while putatively acting as an anti-cancer agent in triple-negative breast cancer patients [105]. Furthermore, AP2B1 is upregulated as a putative defense mechanism against heavy metals [106] while is identified as a target of *influenza A* virus promoting virus internalization [107]. AP2M1, the only identified AP2 middle-sized subunit, controls complex-cargo protein affinity via phosphorylation [108,109], a characteristic that is exploited by viruses such as rabies (RABV) [110], dengue (DENV) [111] and hepatitis C [112] viruses. Moreover, a recent report showed that a wide spectrum of viruses, including coronaviruses , that recently garnered increased attention, have a AP2M1 binding motif YxxØ for viral infection [113]. In addition to other subunits, AP2M1 is important in ECM development [114] and its aberrant expression has pathological consequences, such as the development of autism [115] and Parkinson’s disease, in neural tissues [116]. The role of AP2M1 in signal transduction has been reported in several studies. In fact, its endocytosis function is required for maintaining dendrite cell polarity [117]; restraining signals, such as insulin signal responding glucagon-like peptide receptor, by downregulating receptor presentation on the cell surface [118]; or controlling endosomal trafficking of transcription factors, such as EGFR, to the nucleus [119]. These functions have an effect on cancer cells, as AP2M1 regulates insulin-like growth factor-1 receptor (IGF1R) in prostate cancer [120] and EGFR internalization in bladder cancer [121]. AP2M1 is also related to chemoresistance in AML [122], is upregulated in adenoid cystic carcinoma and mucoepidermoid carcinoma [123], and is a putative biomarker for hepatocellular carcinoma [124]. On the other hand, AP2M1 exhibits anti-tumor activity by inhibiting autophagic activity in acute lymphoblastic leukemia [125]. Controlling the expression of AP2M1 was proposed as a potentially effective treatment for cystic fibrosis, a genetic disorder caused by defective cellular trafficking [126], demonstrating its potential as a target gene for diverse diseases. Finally, the small subunit AP2S1 was shown to be critical for cellular calcium-sensing receptor activity [127], and AP2S1 mutations were reported to result in familial hypocalciuric hypercalcemia type 3, which causes high calcium levels in bloodstream [128]. Most studies involving AP2S1 were linked to this genetic disease [129–135], with  one exception linking AP2S1 to obesity [88],  which suggests that its major function is in calcium-sensing pathway.

## 4. AP3 complex

AP3 main function is known to be trafficking between endosomes, lysosomes, lysosomes related organelles (LROs) and synaptic vesicles (Figure 1) [136]. It was first identified in rats with two isoforms of the medium μ subunit: one was ubiquitously expressed (μ3A), while the other was specifically expressed in brain tissue (μ3B) [16]. The large β subunit is also expressed in two isoforms, with one ubiquitously expressed (β3A) [137] and the other being neural cell-specific (β-NAP or β3B) [138]. The two identified AP3 small σ subunits are both ubiquitously expressed [139] and interact with the large subunit δ ear domain, leading to a conformational change in the AP3 complex that regulates its recruitment to the membrane [140]. Unlike AP1 and AP2 knock-down [60,141], AP3 knockdown is not embryonically lethal, with the most prominent phenotype being abnormal LRO formation [142]. Although its knockout is not deleterious, AP3 plays an important role in development by affecting Notch signaling [143]. In cells, AP3 was shown to be involved in melanocyte trafficking [144], immune defense by CD1b trafficking [145] and in movement of lytic granules of T cells [146]. Most of AP3 variants in human patients are linked to Hermansky-Pudlak disorder type 2 (HPS2) [147–150], in addition to be involved in other mental disorders such as schizophrenia [151]. The AP3 budding scission system is dynamin 2 independent [152] and is involved in the trafficking of specific proteins such as LAMPI/II [153]. It specifically interacts with the HIV viral Gag protein in endosomes [154]. AP3D1 was first identified from the expressed sequence tag (EST) database through its homology wit the AP1 and AP2 subunits [155]. AP3D1 mutation in its *Drosophila* ortholog causes defective eye pigmentation phenotype due to dislocation of pigment granules [156]. In humans, AP3D1 has been identified as a candidate gene for chondrogenesis [157]. As the only known δ subunit of AP3, variation in human AP3D1 is usually linked to HPS2, with few examples that have variable phenotypes; for instance, HPS10, which shows symptoms reminiscent of HPS2, in addition to neurological defects [158,159]. The mouse model for HPS, the ‘mocha’ mouse, has a defect in the mouse AP3 δ subunit [160]. This mutation is known to affect retinal cell development [161] and hippocampal LRO sorting [160,162]. In addition, aberrant AP3D1 expression is associated with high risks for several cancers [163,164], heart attack [165] and may be involved in chronic obstructive pulmonary disease through its binding to TGF bta2 [166]. The AP3D1 subunit is also a target for bovine leukemia virus gp51 [167], is involved in  HIV viral particles [168] and surface coat proteins [169] release. These findings imply that similar to AP1 and AP2, AP3D1 is also targeted by viruses. AP3D1 has a positive effect in cancer, as it inhibits chemotherapy resistance in colorectal cells by maintaining signal integrity in tumor cells by preventing IFNGR1 lysosomal sorting [170]. Additionally, AP3D1 and all AP3 subunits are downregulated in cervical carcinoma [171], which suggests that AP3 might play a role in the cell-inherent anti-cancer pathway. The ubiquitously expressed AP3B1 [137] is involved in lysosome positioning, as demonstrated by its knockdown [172]. Its deficiency has been reported to cause the deletion or decrease of the whole AP3 complex, leading to HPS2 [148,173–175]. Further, its deletion mouse model, the ‘pearl’ mouse, displays HPS2, night blindness [176], deficient pigmentation in eyes [177] and uteri hypoxia [178]. In addition, pearl mice cells show defects in platelet granules [179]. In human, AP3B1 variants were detected in patients with bleeding diathesis, presumably caused by platelet disorder [180]. AP3B1 also interacts with multidrug resistance protein 4 (MRP4) [181] and is involved in vesicle-associated membrane protein 8 (VAMP8) trafficking and Weibel-Palade body maturation [182] which are crucial processes in platelet function. AP3B1 mutation was reported to cause neutropenia, a deficiency in white blood cells in humans, as well as canine cyclic hematopoiesis in dogs; however, this mutation only resulted in decreased hematopoietic progenitor and decreased granulocyte mobilization upon signaling in pearl mice [183]. Moreover, although hemophagocytic lymphohistiocytosis (HLH) patients, which show excessive immune activation, are reported to have mutations in AP3B1 [184,185], HPS patients or pearl mouse models rarely display HLH symptoms [186,187], indicating that AP3B1 variation might have different effects depending on the mutation and/or species affected. Recently, bioinformatic analysis revealed the putative role of AP3B1 in amyotrophic lateral sclerosis pathogenesis, a neurodegenerative disease that affects muscle movement [188] Alveolar epithelial cells with defective AP3B1 were found to display abnormal mitochondrial formation [189], while lung tissues showed increased matrix metalloproteinase activity [190], suggesting a potential role of AP3B1 in lung tissue. AP3B1 mutation was found to affect natural killer (NK) and NKT cell granule-associated protein release, which presumably explains the susceptibility to infection and lymphoma among HPS2 patients [191]. The cells of HPS patients exhibit less HIV-1 viral particle release [192] presumably due to the interaction of AP3B1 with Kif3A, which is modulated by IP7-mediated pyrophosphorylation [193]. AP3B1 interacts with Nipah, Hendra [194], and SARS-CoV2 virus protein [193], which suggests that its wild-type version is a target of viral infection. AP3B1 expression is controlled by the anti-tumor microRNA-9 in breast cancer [195] and its copy number increases in patients with neurofibromatis type I, an autosomal dominant disorder causing tumor development [196]. Such findings indicate the potential of AP3B1 as a target in cancer treatment. The neuron-specific AP3B2 [139] was first identified as β-NAP [138]. While both ubiquitously expressed AP3B1 and brain specific AP3B2 are expressed in the brain, their roles are non-redundant [197]. AP3B2 is reported to be involved in neural development [198] and proteins sorting of neural lysosomes and of lysosome-related organelles [199]. Further, aberrant AP3B2 was reported to be involved in neural development disorder [200], autoimmune cerebellar ataxia, various extracerebellar symptoms [201] and recently in Alzheimer’s disease [202]. Interestingly, while AP3B2 is reported to be neural specific, it was identified in a screening related to sporadic Hirschsprung disease, a colon developmental disease [203]. Its abberant expression in other tissues was observed as it is a putative biomarker for rectal carcinoma [204]; and possibly involved in skin tropism of melanoma cells [205]. Such findings suggest that AP3B2 might play a developmental role in non-neural tissues, and that its aberrant expression in other tissues might be involved in cancer, putatively in the metastasis mechanism. Only few reports have been published on the two middle-sized AP3 subunits, AP3M1 and AP3M2. In addition to the AP1 and AP2 middle-sized subunits, the ubiquitously expressed AP3M1 was reported to be a target for HIV infection [206]. Neural tissue-specific AP3M2 affects IL-6 levels in astrocytes [207] and clathrin-mediated endocytosis via its phosphatidylinositol (4,5)-bisphosphate binding domain [208]. To our knowledge, no study has reported a significant correlation between the AP3B2 variant and neurological disease. AP3S1 is involved in melanocyte pigmentation [209] and might control insulin receptor substrate-1 subcellular localization in adipocytes [210]. In addition to the fusion mutations detected in colorectal cancer cell lines [211], variants of AP3S2 are associated with diabetes [212,213], a high risk for liver cancer [214] and cardiovascular disorders [215].

## 5. AP4 complex

Although its exact functions are not well-defined, mutation of AP4 in various subunits leads to mental deficiency, including hereditary spastic paraplegia (HSP), mental disability, and Alzheimer’s disease [216–220], which might be partially due to autophagy serving as a key process in neurons [221] and axonal AGT9 sorting deficiency leading to deficient neuron development and maturation [222]. In addition to its reported role in autophagy, it is also reported that AP4 is involved in basolateral transport of epithelial cells (Figure 1) [22]. AP4E1 mutation has been reported to be involved in neural diseases, including cerebral palsy, HSP, and stuttering [223–229]. In mouse testes, it was shown that along with AP1S3, it increases upon fluoride-induced stress [230], and in Arabidopsis, to be involved in the hypersensitive cell death pathway [231]. Along with the fact that it is ubiquitously detected [6,7], such findings suggest that it might have an additional role in other tissues. AP4B1, the other large subunit of AP4, is known to have a binding domain for Tepsin in its C terminal [21]. However, its deletion is not sufficient to abolish Tepsin binding to AP4, suggesting that an additional binding domain exists in the AP4 complex [232]. Similarly to AP4E1, AP4 mutation was reported to affect neural cells, as variants were identified in HSP patients [233–236], and might cause intellectual disability [237]. Although AP4 lacks clathrin-binding domain, AP4M1 was reported to be localized in TGN with clathrin vesicles [19], which might be partially explained by the presence of a portion of AP4 co-localized with AP1 in the kidney epithelial cell line MDCK [22]. Interestingly, AP4M1 is involved in basolateral vesicle transport and co-localizes with cation-independent mannose 6-phosphate receptor (CI-MPR), but not with the transferrin receptor lysosomal-associated membrane protein-2 (LAMP-2) in MDCK cells [22]. In HeLa cells, AP4M1 reportedly interacts with LAMP-2 during intracellular transport between endosomes and lysosomes independent of the plasma membrane [238], suggesting that AP4 is involved in independent intracellular transport mechanisms based on their partner proteins. AP4M1 is upregulated and redistributed to neuronal axons under oxygen-glucose deprivation stress [239] and variants affect ocular development [240], are involved in Alzheimer’s disease [241,242], neurodegeneration with brain iron accumulation [243], cerebral palsy [244], and congenital spastic tetraplegia [245]. Knockout of the small subunit AP4S1 in zebrafish resulted in symptoms such as spastic paraplegia 52 [246] and affected immune response in frog [247]. Furthermore, the AP4S1 variant caused iron accumulation and neurodegeneration in human patients [248].

## 6. AP5 complex

AP5 is the most recently identified adaptin complex. Although AP5M1 has a low expression and homology to other subunits, it possess an unique μ homology domain, which led to the discovery of AP5 that is localized in late endosomes and lysosomes and putatively involved in late endosome retrieval (Figure 1)[9]. Similarly to those in AP3 and AP4, defects in AP5 have also been linked to HSP; two of its accessory proteins, SPG11 and SPG11, were identified in HSP cells [249] and were reported to be involved in localization and/or AP5 stability [250]. Although further analysis is required to define the function and location of AP5, one of its roles is to recycle lysosome proteins to the Golgi complex [251]. AP5Z1 variants were reportedly detected in HSP patients [252–254] and were suggested to be partially the cause of mitochondrial defects in axons [255]. In addition, knockout of AP5Z1 causes defection in theretrieval of CI-MPR, GOLIM4, and GOLM1 proteins from endosomes [256]. Moreover, defects in AP5Z1 cause additional symptoms in brain and skin tissue due to abnormal lysosomal material, suggesting that AP5 dysregulation can result in other diseases related to deficient lysosome recycling [257]. In addition, although the specific mechanisms are yet to be discovered, AP5Z1 and the downregulation of AP5M1 were observed in patients with sporadic spastic paraplegia [254] while AP5B1 variants were identified in patients with allergic disease [258–262]. AP5M1 was reported to be involved in cell apoptosis pathways, which are controlled by mitochondrial protein [263–269], suggesting that lysosome recycling might have a broad effect or the AP5 complex might have additional functions in cells.

## 7. Conclusion

Understanding and controlling intracellular trafficking can be crucialaspects in disease treatments. For example, intracellular trafficking can be used by viruses to mediate their entry into host cells or promote their replication [270], a deficiency in intracellular transport can cause severe genetic diseases [271] while it is also putatively a target for treating cancer [272]. Moreover, endocytosis is an important feature that should be considered in drug delivery [273]. Endocytosis affects the presentation of crucial receptor proteins at the cell surface [274]. Indeed, an *in vitro* study using GBM cell lines revealed that the expression of the *μ* subunit of AP5 controlled GBM cell sensitivity to Tumor Necrosis Factor Related Apoptosis-Inducing Ligand, suggesting that AP5 might be involved in the apoptosis signaling pathway triggered by anti-cancer drugs [263]. As demonstrated in this review, extensive work has already been carried out by several groups in this field. Clathrin dependent protein trafficking in the TGN was not only indispensable during development, as it was shown by AP1 knockout mutant, but it was also involved in cancer cell proliferation, while its precise mechanism has yet to be investigated. Deficient AP1 subunits were also linked to defection in neuron cells and pigment misallocation in skin cells, presumably because of aberrant protein sorting in TGN. Impairment of the also developmental necessary AP2, which major role is to control endocytosis from plasma membrane, was reported in diseases with a broad spectrum by its involvement in mechanisms such as endocytic cargo transportion, ECM communication and displaying of receptors at the membrane surface. Further studies will be needed to elucidate involved mechanisms as it was shown to be controlling yet uncurable diseases, such Alzheimer’s disease. Patients case studies has also shown that AP2 was also hijacked by pathogen for infection or cancer cells for controlling receptor display, suggesting that its study can potentially improve further treatments. Interestingly, reports show that some of its tissue specific subunits are expressed in unexpected tissues and/or aberrantly expressed in pathological tissue, suggesting that yet additional roles for AP2 and consequences in diseases are yet to be identified. AP3, which function is to sort proteins in lysosomes, LRO and synaptic vesicles recycling in neural cells is notably linked to HPS2, while also being reported to affect various diseases such as Alzheimer’s disease, autoimmune diseases and melanoma, reflecting the variable impact of aberrant sorting in organelles. AP4 and AP5 precise mechanisms are yet to be defined and therefore pathways involved in known diseases linked to their deficiency such as HSP or Alzheimer’s disease is yet to be identified. Adaptin-related intracellular trafficking pathways are complex and diverse, which can be interfered with based on the few known binding domains and corresponding partner proteins listed in Table 2. Although all known partner proteins are not listed herein, more partner proteins and mechanism are yet to be identified, particularly in the less known AP4 and AP5 complexes. It is notable that while diseases or genetic disorders linked to APs deficiency are various, reflecting the multifaceted role of selective vesicular sorting and transport have in cell function, most of the case studies results are from screening for candidate genes from a list of genes known to be involved to vesicle intracellular trafficking. Hence, most APs variants were detected in patient with diseases such as neuronal disorders, melanosomes missorting or in cohorts with high virus infection susceptibility. Admittedly, proper vesicular trafficking and protein sorting might be specifically critical in those conditions. For example, as neuron cell major function is to receive and transmit signals, mislocation of proteins in synaptic vesicles might result in severe phenotypes, which can be reflected on the fact that all APs variants are shown to be detected in case studies related to neuron defect disease or disorder. However, as mentioned above, all isoforms expressed and binding partners are yet to be discovered, especially for the less well studied AP4 and 5. Moreover, while APs link to diseases and disorders has been identified, their mechanisms have only been partially explained, especially for its role in cancer cell. This suggest more APs linked diseases/disorders are to yet be discovered and show their potential as biomarkers and/or putative target for treatments.

**Table 2.** Motifs and binding partners of AP subunits

Complex	Subunit	Binding partner	Recognizing motif
AP1	γ	Rabaptin-5, γ-synergin, enthoprotin/Clint/epsinR, p56 in mammalian cells, Ent3p and Ent5p in yeast [275], Clathrin [276]	ΨG(P/D/E)(Ψ/L/M), Ψ is an aromatic residue [275]; LLDLL [276]
	β1	Clathrin [277]	LLNLD [277]
	μ1	HIV-Nef [278]	YXXØ, X is any amino acid, Ø is a bulky hydrophobic amino acid [270,279]; EXXXLL [278]
	σ1		DXXLL [270,279]
AP2	α	Eps15 [280], epsin 1, amphiphysin I, AP180 [281], auxilin, dynamin 1 and 2, SJ170 [282], stonin2 [283], Clathrin [276]	DPW; DPF [280]; FXDXF [281]; WXXF [282]; LLDLL [276]; WVXF [283]
	β2	Clathrin [277]	LLNLD [277]
	μ2	HIV-Nef [278], stonin2 [283]	YXXØ, [270,279]; EXXXLL [278]; WVXF [283]
	σ2		DXXLL [270,279]
AP3	δ	Clathrin?	
	β3	Clathrin [12]	SLLDLDDFN [12]
	μ3	HIV-Nef [278]	YXXØ [270,279]; EXXXLL [278]
	σ3		DXXLL [270,279]
AP4	ε		
	β4		
	μ4	CI-MPR [19], LAMP-2 [238], Alzheimer’s Disease precursor protein (APP) [241]	YXXØ [270,279]; FYD(P/R)F; HTGYEQF [238]; YKFFE [241]
	σ4		DXXLL [270,279]
AP5	ζ		missing LLDLL binding domain [9]
	β5		
	μ5		alteration of YXXØ binding domain [9]
	σ5		
